# Prevalence and characters of post-acute COVID-19 syndrome in healthcare workers in Kashan/Iran 2023: a cross-sectional study

**DOI:** 10.1186/s12912-024-01733-2

**Published:** 2024-03-20

**Authors:** Hamidreza Zeraatkhah, Negin Masoudi Alavi, Hanieh Ziabakhsh, Zahra Mahdaviasl

**Affiliations:** 1 Department of Medical Surgical and Geriatric Nursing, Kashan University of medical Science, Kashan, Iran; 2 Trauma Nursing Research Center, Department of Medical Surgical and Geriatric Nursing, Kashan University of medical science, Kashan, Iran; 3 Kashan Faculty of Nursing and Midwifery, Kashan University of Medical Sciences, Ghotb Ravandi Highway, Kashan, Iran

**Keywords:** Long COVID-19, Health personnel, Associated factors

## Abstract

**Background:**

Post-acute COVID-19 syndrome that is called long COVID-19 consists of the symptoms that last more than 12 weeks with no other explanation. The present study aimed to determine the prevalence, frequency of symptoms, and risk factors of long COVID-19 in the healthcare workers (HCWs) of a selected hospital in Kashan/Iran in 2023.

**Methods:**

A total of 350 HCWs with a history of COVID-19 infection were randomly recruited to the study from February to May 2023. Participants completed a questionnaire including demographic characteristics, information related to COVID-19 infection, underlying diseases, and a checklist of long COVID-19 symptoms. Mann‒Whitney U test, chi-square test, T‒tests, and binary logistic regression were used for data analysis by SPSS 16.

**Results:**

The results showed that 75.7% of HCWs experienced symptoms of long COVID-19. The most common symptoms were fatigue (53.1%), cough (43.1%) and muscle weakness (37.1%). In bivariate analysis job title, body mass index (BMI), frequency and number of symptoms of COVID-19 infection, preexisting disease, tobacco use, age, and years of experience showed a significant statistical association with long COVID-19. In binary logistic regression the number of symptoms during COVID-19 infection, nursing occupation, use of corticosteroids, and symptoms of dyspnea and loss of taste could explain the occurrence of long COVID-19.

**Conclusion:**

The long COVID-19 is a prevalent condition among HCWs especially nurses. Symptoms of long COVID-19 such as fatigue and cough can persists over time. This chronic condition has significant associations with different clinical risk factors.

**Supplementary Information:**

The online version contains supplementary material available at 10.1186/s12912-024-01733-2.

## Introduction

COVID-19 is a disease caused by SARS-Cov-2 virus started a pandemic that affected all countries in the world [1]. According to the WHO, by the May of 2023, the cumulative number of cases of COVID-19 worldwide stands at 765,222,932, with nearly seven million deaths [2]. In Iran, by the September of 2023, 7,617,762 people have contracted the disease with 146,410 deaths [3].

COVID-19 has a wide range of presentations, from asymptomatic status to several problems, such as fever, chills, cough, fatigue, myalgia, headache, diarrhea, vomiting, dyspnea, anosmia and insomnia [4]. It mainly influences the respiratory system and causes pneumonia, but it can have several extra-respiratory complications, such as thrombosis, coronary syndrome, and hepatic and skin problems [5]. COVID-19, similar to other epidemics of coronaviruses such as SARS in 2003, and MERS in 2012 can have chronic and multiorgan complications [6]. The post-acute COVID-19 syndrome that was later called long COVID-19 was defined as the continuation or development of new symptoms 3 months after the initial SARS-CoV-2 infection, with these symptoms lasting for at least 2 months with no other explanation [7]. The prevalence of long COVID-19 has been reported between 32 and 87% in different studies in general population [8–14]. Besides the wide range of prevalence, the symptoms of long COVID-19 also have been numerous. Fatigue, dyspnea, and cognitive problems that might continue after the disease or start sometime after recovery have been some common symptoms of long COVID-19 [13]. Dyspnea during activity (22.9%) [12], myalgia (88%), and headache (83%) [10] had been reported as other prevalent symptoms. The severity of symptoms might increase or decrease over time [13]. In a study, long COVID-19 was not associated with the severity of the initial disease or hospitalization but was more prevalent in females [14]. Some risk factors for long COVID-19 have been female sex, age, obesity, smoking, and chronic diseases [15–18].

During the COVID-19 pandemic, many professions stopped working or changed their jobs as online carriers; in contrast, the health care workers (HCWs) encountered the maximum workload with long shifts and very hard work conditions with much physical and mental stress [19]. HCWs contracted COVID-19 almost twice as often as general population; some experienced the disease several times with different variants of the virus, with a significant burden [20–22]. Until May 2020, a total of 152 888 COVID-19 infections and 1413 deaths in HCWs were reported worldwide [23]. In a cohort study frontline HCWs had an hazard ratio of 11·6 (95% CI: 10·9 to 12·3) compared with the general community, for reporting a positive COVID-19 test [24].

These factors might influence the prevalence, presentation, and severity of long COVID-19 in HCWs, while there are limited studies about this subject. Besides long COVID-19 can negatively affect the quality of HCWs services. Fatigue as a common symptom of long COVID-19 is negatively associated with nursing performance, patient-safety, and organizational outcomes [25]. These potential consequences make this problem more important in HCWs. The current study aimed to determine the prevalence, frequency of symptoms, and risk factors of long COVID-19 in the healthcare workers (HCWs) of a selected hospital in Kashan/Iran in 2023.

## Materials and methods

### Study design and population

This cross-sectional study was carried out in Shahid Beheshti Hospital in Kashan/Iran from February to May 2023, according to STROBE checklist. This hospital is the only general governmental hospital in Kashan Province, with 740 beds that provides health care services to 400,000 residents. During the pandemic, this hospital was the center for providing inpatient services to COVID-19 patients. In Iran, the first confirmed case of COVID-19 was detected in 19 February, 2020, in Qom province, that is the neighbor city of Kashan. Until May 2023 that WHO declared the end of COVID-19 as public health emergency of international concern, 430,000 have treated for COVID-19 in Kashan province, and 21,560 patients have hospitalized for the disease. Six waves of the disease have been detected in the region. This study has been done after the outbreaks, when the COVID-19 was not a health concern and limitations of the disease had been lifted.

The health personnel with maximum engagement in treatment of COVID-19 patients during pandemic, including physicians, nurses, physiotherapists, laboratory and radiology technicians were recruited to the study by stratified random sampling based on their job. Considering the prevalence of long COVID-19 to be 32% [12], a confidence level of 95%, and an error of 5%, the sample size with the formula (Z^2^pq/d^2^) was calculated to be 350. The inclusion criteria were working as a health care provider during COVID-19 pandemic for at least 6 months, having a history of COVID-19 according to positive PCR, chest CT-scan, or diagnosis by an infectious disease specialist, and willingness to participate in the study. The names of the HCWs were listed alphabetically, and stratified random sampling, according to the job was performed. If a subject did not want to participate, the next person in the list was substituted.

### Questionnaire and data gathering

After extensive literature review a questionnaire was developed by research team (Supplementary file 1) that had two parts: The first part was about attributed factors of demographics (including, age, sex, job, and BMI), the variables of the COVID-19 (the number of times a person has been infected with COVID-19 during the pandemic, the symptoms during the disease, hospitalization, the drugs used for treatment and vaccination), and the history of other diseases. In the second part, the 30 possible symptoms of long COVID-19 according to the literature were listed. The participants could choose the symptoms that they were experiencing for at least 2 months after COVID-19 infection with no other explanations; they also could rate the severity of the symptoms from 1 to 10. Participants could write down any other symptoms that were not in the list. The content validity of the questionnaire was qualitatively investigated by 12 experts in nursing and infectious diseases. They suggested some changes that were made to the questionnaire. The content validity of the final questionnaire was approved by the experts. 25 nurses completed the questionnaire without any problem in understanding or answering the questions, same nurses completed the questionnaire after 2 weeks, and the intra-class correlation coefficient (ICC) was calculated 0.925, which showed acceptable reliability. HCWs completed the questionnaires in the hospital, during the rest time, or just after the working shift. They were given adequate time to complete the questionnaire and the time of the COVID-19 infection of some respondents was checked with their sick leave to assess the potential recall bias.

### Statistical analysis

Descriptive statistics were used to show the demographic characteristics of the participants, long COVID-19 prevalence and its symptoms. The Kolmogorov-Smirnov test was used to assess the normality of the variables such as age and BMI. The statistical tests of chi-square, and Mann-Whitney U were used to investigate the association between long COVID-19 and attributed factors. A binary logistic regression model was used to identify risk factors for long COVID-19, and odds ratios (ORs) with associated 95% confidence intervals were calculated. A p value of ≤ 0.05 was considered statistically significant. SPSS (version 16, SPSS Inc., Armonk, NY, USA) was used to carry out the analyses.

### Ethical considerations

This study was approved by the ethical committee of the research deputy of Kashan University of Medical Sciences, with ethical code IR.KAUMS.NUHEPM.REC.1401.086. The objectives of the study were explained to the participants, and they signed the informed consent form. The confidentiality of the participants was respected in the study. This Research has been performed in accordance with the Declaration of Helsinki.

## Results

A total of 350 HCWs took part in the study. The mean age of the participants was 34.4 ± 8.6 years (Range 23–77), 194 (55.4%) were women, and 247 (70.6%) were nurses. The mean BMI was 25.1 ± 3.8. The characteristics of the participants are presented in Tables 1 and 2. The participants reported an average of 6.8 ± 2.9 symptoms (range 1–16) during COVID-19 infection and the most common symptoms were fever (76.9%), fatigue (68.9%), and cough (68.9%). A total of 265 (75.7%) HCWs reported long COVID-19 symptoms, and 85 (24.3%) did not have any remaining symptoms. The most common symptoms of long COVID-19 were fatigue (53.1%), cough (43.1%), muscle weakness (37.1%), and hair loss (30.6%) (Table 3). In bivariate analysis, long COVID-19 showed a significant statistical association with job, BMI, frequency of COVID-19 infection, preexisting disease, tobacco use, age, work experience, and number of symptoms during COVID-19 infection (Tables 1 and 2).

**Table 1:** The characteristics of the study population and their association with long covid-19 (*N* = 350)

Variables		N (%)	Long Covid-19 N (%)	Z	P value
Sex	Female	194 (55.4%)	149 (76.8%)	0.128^a^	0.596
	Male	156 (44.6%)	116 (74.4%)		
Job	Nurse	247 (70.6%)	204 (82.6%)	29.829 ^b^	0.000^*^
	Nurse assistants	15 (4.3%)	13 (86.7%)		
	Physician	72 (20.6%)	37 (51.4%)		
	Physiotherapist	4 (1.1%)	2 (50%)		
	Laboratory technician	6 (1.7%)	5 (83.3%)		
	Radiology technician	6 (1.7%)	4 (66.7%)		
BMI	Under and normal weigh (< 19-24.9)	182 (52%)	145 (79.7%)	7.249^a^	0.027^*^
	Pre-obesity (25.0–29.9)	135 (38.6%)	92 (68.1%)		
	Obesity (≥ 30.0)	33 (9.4%)	28 (84.8%)		
Vaccination	Yes	320 (91.4%)	241 (75.3%)	0.328^a^	0.661
	No	30 (8.6%)	24 (80%)		
Frequency of covid-19 infection	Once	182 (52%)	136 (74.7%)	16.626^a^	0.005^*^
	Twice	109 (31.1%)	73 (67%)		
	Three times	49 (14%)	46 (93.9%)		
	More than 3 times	10 (2.9%)	10 (100%)		
Hospitalization due to covid-19	yes	45 (12.9%)	38 (84.4%)	2.14^a^	0.143
	No	305 (87.1%)	227 (74.4%)		
Hospitalization in ICU	Yes	16 (4.6%)	15 (93.8%) 250 (74.9%)	2.966^a^	0.085
	No	334 (95.4%)			
Pre-existing Disease	Yes	59 (16.9%)	52 (88.1%)	5.954^a^	0.015^*^
	No	291 (83.1%)	213 (73.2%)		
Tobacco use	Yes	26 (7.4%)	24 (92.3%)	4.206^a^	0.040^*^
	No	324 (92.6%)	241 (74.4%)		
Corticosteroid usage	Yes	129 (37%)	116 (89.9%)	22.42^a^	0.000^*^
	No	221 (63%)	149 (67.4%)		

^a^ The z values of Pearson Chi-square test

^b^ The z values of Fisher’s Exact test

^*^ The significant association

**Table 2:** The association of long covid-19 and numeric variables

Variables	Mean ± Sd	Long covid-19		Z^b^	P value
		Yes	No		
Age (Years)	34.4 ± 8.6	33.6 ± 8.3	36.9 ± 9	-3.209	0.001^*^
Work experience	11.7 ± 8	13.3 ± 8	11.1 ± 7.9	-2.366	0.018^*^
The number of symptoms during covid-19 infection	6.8 ± 2.8	7.3 ± 2.9	5.2 ± 2	-6.024	0.0001^*^
BMI	25.1 ± 3.8	25 ± 4	25.4 ± 3.3	-1.244	0.213

^b^ The Z values of Mann-Whitney U test

^*^ The significant association

**Table 3:** The frequency of long covid-19 symptoms and their severity

Symptoms	No	%	The severity of symptoms in 1 to 10 scale
Fatigue	186	53.1	5.1 ± 2.3
Cough	151	43.1	4.7 ± 2.4
Muscle weakness	130	37.1	5.6 ± 2.4
Not feeling well	120	34.3	4.4 ± 2.4
Hair loss	107	30.6	5.6 ± 2.7
Myalgia	97	27.7	5.1 ± 2.5
Joint pain	90	25.7	5.6 ± 2.4
Headache	88	25.1	5 ± 2.8
Anosmia	85	24.3	6.9 ± 3
Sensitivity in throat	83	23.7	4.5 ± 2.4
Anorexia	82	23.4	5.3 ± 2.3
Loss of taste	78	22.3	6.5 ± 2.7
Dyspnea during activity	74	21.1	4 ± 1.9
Change of voice	74	21.1	5 ± 2.7
Confusion	63	18	5.6 ± 2.1
Chest pain	54	15.4	4.5 ± 2
Anxiety	53	15.1	4.8 ± 2.4
Memory loss	51	14.6	4.3 ± 2.2
Tachycardia	46	13.1	4.6 ± 2.4
Insomnia	45	12.9	6.5 ± 2.5
Depressive mood	44	12.6	4.8 ± 2.7
Vertigo	43	12.3	4.5 ± 2.4
Loss of concentration	41	11.7	4.4 ± 2.6
Dyspnea during rest	38	10.9	3.3 ± 2
Fever	35	10	5.5 ± 3.1
Xerostomia	32	9.1	4.2 ± 2.2
Unpleasant smell	31	8.9	6.1 ± 3.4
Decreasing libido	22	6.3	5 ± 3
Abdominal pain	20	5.7	2.9 ± 2.1
Skin rash	14	4	5 ± 3.5

Considering long COVID-19 as the dependent variable, all possible independent variables including symptoms during COVID-19 infection, were entered into binary logistic regression. The variables could explain 28 to 42% of long COVID-19 occurrences. From all the variables, the number of symptoms during COVID-19 infection (odds ratio = 1.43, 95% CI 1.191–1.727), nursing occupation (odds ratio = 2.52, 95% CI 0.321–19.894), use of corticosteroids (odds ratio = 4.07, 95% CI 1.887–8.796), symptoms of cough (odds ratio = 0.089, 95% CI 0.089–0.432), dyspnea (odds ratio = 2.49, 95% CI 1.19–5.241), loss of taste (odds ratio = 6.2, 95% CI 2.283–16.808), and fatigue (odds ratio = 0.485, 95% CI 0.224–0.965) could explain the long COVID-19 significantly (Table 4). The HCWs who had fatigue and cough during the COVID-19 infection experienced less long COVID-19; on the other hand, the number of symptoms during initial COVID-19 infection, specially dyspnea and loss of taste, receiving corticosteroid during infection and nurses reported more frequency of long COVID-19.

**Table 4:** The results of binary logistic regression- long covid-19 as dependent variable

Independent variables	B	Wald	Sig	Odds Ratio	95% CI^c^ for Odds Ratio	
					Lower	Upper
Job (Nursing)	0.927	24.678	0.000	2.527	0.321	19.894
Number of Symptoms	0.360	14.439	0.000	1.434	1.191	1.727
Corticosteroid	1.405	12.792	0.000	4.074	1.887	8.796
Cough	-1.629	16.309	0.000	0.196	0.089	0.432
Dyspnea	0.915	5.858	0.016	2.497	1.19	5.241
Fatigue	-0.723	4.251	0.039	0.485	0.224	0.965
Loss of taste	1.824	12.819	0.000	6.194	2.283	16.808

^c^ Confidence Interval

## Discussion

The prevalence of long COVID-19 was 75.7% in HCWs. In a study by Peters and colleagues in Germany, 73% of health and social services workers experienced persistent symptoms for more than three months [26], which is comparable to our study. The prevalence of long COVID-19 in health care workers has been 45% in England [27], 27.4% in Brazil [28], 47.4% in Morocco [29], and 30.34% in India [30]. The prevalence of long COVID-19 in the general population has been 10–20% in England [31] and 22.9% in Switzerland [32]. A meta-analysis estimated the prevalence of long COVID-19 as 42% globally [33], and it was 45.9% in another meta-analysis [34]. According to the current study, it seems that the prevalence of long COVID-19 is higher in HCWs than in the general population.

In the current study, the most common symptoms of long COVID-19 were fatigue, cough, muscle weakness, and hair loss. Almost all studies have reported fatigue as the most common symptom of long COVID-19 [34–37]. In a meta-analysis, the prevalence of dyspnea (18%), arthromyalgia (26%), and insomnia (12%) [36] were almost the same as the current study. Dyspnea as a common symptom had a pooled prevalence ranging from 35 to 60% in one study [34], which was higher than the 21% in current study. In some studies, the prevalence of long COVID-19 symptoms seems to be quite different, such as fatigue in 6.8% [38] and chest pain in up to 89% of patients [35], compared to 53% and 15%, respectively in the current study. Depression, memory loss, and concentration difficulties were less frequently reported in the current study than in the meta-analysis [36]. The instrument of reporting the symptoms and the time interval between COVID-19 infections can influence the type and the prevalence of the symptoms, so the comparisons should be done cautiously, although the pool of the symptoms seems to be similar in different studies.

The symptoms during the initial COVID-19 infection seem to be related to long COVID-19. In the current study, the patients who had more symptoms during infection and experienced the symptoms of dyspnea and loss of taste had a higher, and those with symptoms of fatigue and cough had a lower rate of long COVID-19. Existing evidence suggests that those with more severe initial illness are more likely to suffer from sequelae after one year [36]. Sudre et al. reported that individuals with more than five symptoms within the first week of initial illness were at high risk for the development of long COVID-19 and found it to be the single, strongest predictive factor. The initial symptoms of fatigue, headache, dyspnea, hoarseness of voice and myalgia were predictive factors [18]. Ballouz reported that symptoms of altered taste or smell, postexertional malaise, fatigue, dyspnea, and reduced concentration and memory had the highest excess risks for the development of long COVID-19 [32]. Dyspnea and loss of taste in the initial infection in this study, along with other studies, seem to be predictive factors for long COVID-19, although contrary to other studies, we did not find fatigue as a risk factor.

Some studies have shown that long COVID-19 is more prevalent in females [35, 36, 39]. A meta-analysis also confirmed that female sex was associated with long COVID-19 symptoms [40]. We did not find such an association in our study. The results regarding the association between sex and long COVID-19 are contradictory, and some studies support this association, whereas others do not [18, 41, 42]. Age is another risk factor. A meta-analysis did not reveal an association between old age and long COVID-19 [40]. However, some single studies showed a higher prevalence of long COVID-19 in older adults [35, 43]. In the current study, the binary logistic regression did not reveal such an association.

There are many contradictory results about the risk factors for long COVID-19; for example, some studies reported medical comorbidities as risk factors [40, 44], and some did not [37]. It is also true about vaccination and hospitalization or using mechanical ventilation in treatment [29, 37].

The long COVID-19 was statistically more prevalent in nurses and nurse assistants compared to physicians. A study in Italy revealed the negative effects of the pandemic on healthcare professionals and especially among nursing staff that reported worse condition of sleep disturbances, anxiety, and depression compared to physicians after two years [45]. These consequences might affect the quality of services of HCWs, specially nurses, that have not been investigated adequately.

This study has some limitations, first the HCWs were under significant work-related stress during COVID-19 outbreak, and the symptoms of long COVID-19 are non-specific, so the symptoms of occupational burnout cannot be differentiated from the symptoms of long COVID-19. Second this was a survey relying on the subjective experiences of the respondents, so there might be variation between participants about how they feel the existence of the symptoms and their severity.

## Conclusion

This study showed that long COVID-19 is a prevalent and serious condition among HCWs especially nurses, who are the largest group of healthcare professionals. This chronic condition may influence the quality of services that HCWs provide within the health systems. This is an important issue that needs further investigation in future studies. Besides there is a need for effective treatment strategies for controlling the symptoms of long COVID-19. Health system needs to manage the long-term physical and mental health adverse consequences of COVID-19 pandemic in HCWs, specially nurses that endured high work pressure during pandemic.

### Electronic supplementary material

Below is the link to the electronic supplementary material.

Supplementary Material 1

## Data Availability

The datasets used and/or analysed during the current study are available from the corresponding author on reasonable request.

## Declarations

This study has been done according to the STROBE guideline. The Declaration of Helsinki has been respected in this study.

### Authors’ information

This section is optional.

## Abbreviations

BMI: Body mass index
CT SCAN: Computed Tomography Scan
HCW: Health Care Workers
MERS: Middle East Respiratory Syndrome
PCR: Polymerase Chain Reaction
SARS: Severe Acute Respiratory Syndrome
SARS-COV2: Severe acute respiratory syndrome coronavirus 2
WHO: World Health Organization
