# Supplementary material for: Prevalence and characters of post-acute COVID-19 syndrome in healthcare workers in Kashan/Iran 2023: a cross-sectional study
Source: BMC Nurs. 2024 Mar 20;23:186. doi: 10.1186/s12912-024-01733-2 (PMC10953126; doi:10.1186/s12912-024-01733-2)
Supplement: Supplementary file 1 — Supplementary Material 1 [file 12912_2024_1733_MOESM1_ESM.docx]

Dear Colleague

This questionnaire has been designed to study the long COVID-19 and its attributed factors in health care workers. Long COVID-19 is the continuation or development of new symptoms 3 months after the initial COVID-19 infection, with these symptoms lasting for at least 2 months with no other explanation. Please read the questions carefully and choose the best answer.

Thank you for your time.

| The Attributed Factors | | | |
| --- | --- | --- | --- |
| Date: | Age: | Sex: male Female | Weigh: Height: |
| Occupation: Nurse Nurse Assistants Physician Physiotherapist Laboratory Technician Radiology Technician | | | |
| Education: Diploma Technician Bachelor degree Master degree GP MD | | | |
| How long have you worked with COVID-19 patients? Year Month | | | Department: |
| How many times did you get the COVID-19? Once Twice Three times More: | | | |
| How did you diagnosed with COVID-19 last time you get the disease?  Positive PCR Lung involvement in CT scan The specialist diagnosis | | | |
| The date of the last diagnosis: Year/Month/Day | | | |
| Have you hospitalized for the COVID-19? Yes No How many times: How many days: | | | |
| Have you hospitalized for the COVID-19 in ICU? Yes No How many times: How many days: | | | |
| Do you have any pre-existing disease: Yes No  Please specify the kind of the disease: Cardiac Hypertension Diabetes Respiratory  The immunodeficiency problems Neuro-psychiatry problems Others: | | | |
| Are you smoking: Yes No | | | |
| What was your symptoms the last time you got the COVID-19? You can choose more than 1 symptom.  Fever Runny nose Fatigue Chill Cough Dyspnea Diarrhea Anosmia Voice Change Loss of appetite Loss of taste Exertional fatigue  Other symptoms: | | | |
| The long COVID-19 Questionnaire | | | |
| Please specify the symptoms that you had not before COVID-19, but started after COVID-19 and lasted for at least 2 months. For every symptom show the severity with 0 to 10 scale that 0 shows no symptom and 10 shows the very severe symptoms. | | | |
| 1. Dyspnea during rest: Yes No The severity (0-10):  Dyspnea during activity : Yes No The severity (0-10): | | | |
| The severity 0-10 (0 means no dyspnea and 10 means sever dyspnea, that you cannot talk properly because of dyspnea): | | | |
| 2. Cough: Yes No The severity (1-10): | | | |
| 3. Fatigue: Yes No The severity (1-10): | | | |
| 4. Skin rash: Yes No The severity (1-10): | | | |
| 5. Muscle weakness: Yes No The severity (1-10): | | | |
| 6. Not feeling well: Yes No The severity (1-10): | | | |
| 7. Hair loss: Yes No The severity (1-10): | | | |
| 8. Myalgia: Yes No The severity (1-10): | | | |
| 9. Joint pain: Yes No The severity (1-10): | | | |
| 10. Headache: Yes No The severity (1-10): | | | |
| 11. Anosmia: Yes No The severity (1-10): | | | |
| 12. Sensitivity in throat: Yes No The severity (1-10): | | | |
| 13. Anorexia: Yes No The severity (1-10): | | | |
| 14. Loss of taste: Yes No The severity (1-10): | | | |
| 15. Change of voice: Yes No The severity (1-10): | | | |
| 16. Confusion: Yes No The severity (1-10): | | | |
| 17. Chest pain: Yes No The severity (1-10): | | | |
| 18. Anxiety: Yes No The severity (1-10): | | | |
| 19. Memory loss: Yes No The severity (1-10): | | | |
| 20. Tachycardia: Yes No The severity (1-10): | | | |
| 21. Insomnia: Yes No The severity (1-10): | | | |
| 22. Depressive mood: Yes No The severity (1-10): | | | |
| 23. Vertigo: Yes No The severity (1-10): | | | |
| 24. Loss of concentration: Yes No The severity (1-10): | | | |
| 25. Dyspnea during rest: Yes No The severity (1-10): | | | |
| 26. Fever: Yes No The severity (1-10): | | | |
| 27. Xerostomia (moth dryness): Yes No The severity (1-10): | | | |
| 28. Unpleasant smell: Yes No The severity (1-10): | | | |
| 29. Decreasing libido: Yes No The severity (1-10): | | | |
| 30. Abdominal pain: Yes No The severity (1-10): | | | |
| Please write any other symptoms that are not in the list: | | | |
